# Meteorin-like protein elevation post-exercise improved vascular inflammation among coronary artery disease patients by downregulating NLRP3 inflammasome activity

**DOI:** 10.18632/aging.205268

**Published:** 2023-12-04

**Authors:** Jingjin Liu, Liwei Diao, Weiyi Xia, Xiaoyi Zeng, Wen Li, Jieru Zou, Tiansheng Liu, Xinli Pang, Yongshun Wang

**Affiliations:** 1 Department of Cardiology, Shenzhen People’s Hospital (The Second Clinical Medical College, Jinan University, The First Affiliated Hospital, Southern University of Science and Technology), Luohu, Shenzhen 518020, Guangdong, China; 2 Shenzhen Clinical Research Center for Geriatrics, Shenzhen People’s Hospital, Luohu, Shenzhen 518020, Guangdong, China; 3 Center for Cardiovascular Disease Prevention and Rehabilitation, University of Chinese Academy of Science, Shenzhen Hospital, Guangming, Shenzhen 518107, Guangdong, China; 4 Department of Health Technology and Informatics, The Hong Kong Polytechnic University, Hong Kong 999077, Hong Kong SAR, China

**Keywords:** coronary artery disease, meteorin-like protein, moderate intensity continuous training exercises, NLR family pyrin domain containing 3 inflammasome, inflammatory cytokines

## Abstract

Background: Coronary artery disease (CAD) has become the most common cause of death worldwide. However, the negative effects of CAD are able to be alleviated via exercises, possibly via increased production of meteorin-like protein (Metrnl). In this study, we aim to evaluate the connection between Metrnl production during exercise with lowered CAD risk and severity.

Methods: Two age and gender-matched groups of 60 human patients, one with CAD, and one without were randomly recruited. The CAD group were subjected to continuous training exercises. Mice were exercised by using a treadmill, establishing an animal exercise model. ELISA was used to measure plasma Metrnl and inflammatory factors. To determine the impact of Metrnl on glucose metabolism, oxygen consumption and extracellular acid rates were taken for untreated, palmitic acid (PA)-treated, and PA+Metrnl co-treated human umbilical vein endothelial cells. Western blot was used to measure expression levels for the NLR family pyrin domain containing 3 inflammasome.

Results: CAD patients had lower Metrnl levels compared to non-CAD controls. Furthermore, higher Metrnl levels post-exercise were inversely associated with LDL, inflammatory cytokines, and CAD severity, as well as being positively associated with HDL. Metrnl was able to counteract against PA-induced HUVEC glucose metabolic dysfunction via reducing ROS production, which in turn lowered NLRP3 inflammasome expression, thereby serving as the basis behind the inverse correlation between Metrnl and inflammatory cytokines.

Conclusions: Exercise was able to increase Metrnl production from skeletal muscle among CAD patients, and subsequently improve patient atherosclerosis via counteracting against endothelial metabolic dysfunction and pro-inflammatory activities.

## INTRODUCTION

Coronary artery disease (CAD) has become the most common cause of death worldwide, particularly in developed countries [1]. Its primary cause has been identified as atherosclerosis [1], a complex pathophysiological process long associated with fatty acid accumulation, particularly low-density lipoprotein cholesterols (LDL), and immune cells beneath the coronary artery endothelial cell layer [2]. More recent studies, though, have found that dyslipidemias, stemming from abnormal fatty acid metabolism, and inflammatory processes are also involved in atherosclerosis and subsequent CAD onset [3]. Dyslipidemia has been linked to genetic mutations of lipid-regulating genes, such as proprotein convertase subtilisin/kexin type 9 (PCSK9). In particular, gain-of-function mutations of this gene have been associated with hypercholesterolemia, or abnormally high levels of serum LDL [4]. Abnormal PCSK9 activation, in turn, resulted in increased expression of pro-inflammatory cytokines tumor necrosis factor (TNF)-α and interleukin (IL)-1β [5], as well as C-reactive protein (CRP), in turn activating nuclear transcription factor κB (NF-κB), which downregulates nitric oxide (NO) production. This downregulation subsequently introduces an imbalance in endothelial NO production, which exacerbates oxidative stress and pro-inflammatory cytokine production, all of which culminates in the formation of atherosclerotic plaques [1, 6, 7]. This connection between dyslipidemia, inflammation, and atherosclerosis is further supported by a number of studies demonstrating that PCSK9 inhibition lowered LDL levels and CAD risk [8, 9]. Additionally, another study has shown that a long non-coding RNA (lncRNA), ENST00000416361, was upregulated in CAD patients, compared to healthy ones. Its knockdown was associated with reduced levels of TNF-α and IL-6, as well as sterol regulatory element binding transcription factor (SREBP) 1 and 2, which have been associated with regulating the expression of other genes involved in fatty acid metabolism [10], thus further highlighting the connection between abnormal fatty acid metabolism, inflammation, and CAD development. Further research, however, is still required to fully confirm these linkages between fatty acid metabolism, inflammation, and CAD, particularly with respect to the role of exercise in modulating these CAD contributory factors.

Exercise has been associated with protective effects against CAD, both in terms of reducing its likelihood for occurrence, as well as for rehabilitation strategies [11, 12]. In fact, it has been associated with reduced reactive oxygen species (ROS) and inflammatory cytokine production, though the exact mechanistic basis linking exercise to lowered inflammation in CAD is still unclear [13]. However, recent studies have intriguingly hinted that a novel adipokine, meteorin-like protein (Metrnl), could serve as that “missing link” between exercise, reduced ROS production and inflammation, and subsequent improvement in patient functioning post-CAD. Metrnl is a ~28 kD protein related to the neurotrophic growth factor meteorin [14, 15], and produced in both adipose tissue and skeletal muscle. In fact, increased Metrnl mRNA levels have been observed post-exercise [16, 17]. As an adipokine, it has been found in numerous studies to favor fat metabolism, and has also been observed to modulate immune responses as an anti-inflammatory agent [14, 18]. With respect to CAD, higher Metrnl levels have been associated with lowered risk for the disease in a study of Chinese patients [19]. Furthermore, Metrnl levels negatively correlated with fatty acid levels, such as LDL, and inflammatory cytokine expression, such as IL-1β [19, 20]. The precise nature of the interactions between Metrnl and these pro-CAD factors, however, are still largely unknown, though a connection between exercise, Metrnl, and inhibition of NLR family pyrin domain containing 3 (NLRP3) inflammasome activity have been documented in a recent investigation [21], which could serve as a possible mechanistic basis behind the alleviative effects of Metrnl on CAD.

In this study, we aimed to elucidate the exact connection between exercise, higher Metrnl, and improved functioning among CAD patients. We confirmed that exercises, such as moderate-intensity continuous training (MICT), resulted in increased Metrnl production from skeletal muscle, compared to sedentary individuals, in both human patients and mouse models. Higher Metrnl was associated with lowered CAD onset and severity, which is likely owed to Metrnl serving as an anti-oxidative factor against ROS production, in turn downregulating NLRP3 inflammasome activity and subsequent production of inflammatory cytokines, as well as counteracting against palmitic acid (PA)-induced endothelial glucose metabolic dysfunction *in vitro,* both of which otherwise contribute to the formation of atherosclerotic plaques. All of these actions thus demonstrate the potential for Metrnl to serve as a possible therapeutic approach for improving CAD patient functioning.

## RESULTS

### Characteristics of the study participants

No significant differences were found between control and CAD groups with respect to age, gender, smoking, BMI, systolic and diastolic blood pressures, plus heart rate, incidences of hypertension, diabetes, and chronic obstructive pulmonary disease (COPD), as well as total cholesterol (TC) and triglyceride (TG) levels. Significant differences, though, were present between the 2 patient groups for high (HDL) and low-density lipoprotein cholesterol (LDL), as well as interleukins (IL)-1β, -6, tumor necrosis factor (TNF)-α, C-reactive protein (CRP), and plasma Metrnl levels. In particular, CAD patients had higher levels of LDL, IL-1β, IL-6, TNF-α, and CRP, as well as lower HDL and Metrnl, compared to controls (Table 1).

**Table 1:** Patient characteristics.

**Patient characteristic**	**Control (n=60)**	**CAD (n=60)**	**P-value**
Age	64.0 (61.9-66.1)	62.00 (59.5-64.5)	0.2234
Female (%)	20 (33.3%)	15 (40.0%)	0.3194
Current smoker (n [%])	31(51.7%)	38 (63.3%)	0.1429
Body mass index (kg/m^2^)	23.37 (22.70-24.05)	26.55 (20.13-32.96)	0.3270
Systolic blood pressure (mm Hg)	142.9 (137.0-148.9)	134.6 (128.2-141.0)	0.0585
Diastolic blood pressure (mm Hg)	84.4 (81.5-87.3)	82.8 (80.0-85.6)	0.4261
Heart rate	77.56 (73.0-78.0)	74.69 (71.0-76.0)	0.1482
Hypertension	27 (45%)	22 (36.67%)	0.3599
Diabetes Mellitus	15 (25%)	21 (35%)	0.2371
COPD	5 (8.3%)	8 (13.3%)	0.3829
TC (mmol/L)	4.58 (4.45-4.71)	4.53 (4.40-4.61)	0.5604
HDL (mmol/L)	1.28 (1.23-1.33)	1.16 (1.11-1.21)	0.0005*
LDL (mmol/L)	2.76 (2.59-2.92)	3.41 (3.25-3.56)	<0.0001*
TG (mmol/L)	1.49 (1.42-1.55)	1.52 (1.44-1.60)	0.5088
IL-1β (pg/ml)	5.93 (5.14-6.73)	16.33 (14.19-18.46)	<0.0001*
TNF-α (pg/ml)	9.75 (8.85-10.65)	17.56 (15.25-19.87)	<0.0001*
IL-6 (pg/ml)	17.89 (14.64-21.15)	23.42 (20.26-26.57)	0.0162*
CRP (mg/L)	5.93 (4.84-7.02)	8.88 (7.90-9.854)	<0.0001*
Plasma Metrnl (pg/ml)	411.9 (378.5-445.2)	214.4 (189.4-239.3)	<0.0001*

All values represent medians (95% CI), except otherwise noted. *P<0.05 was statistically significant. CAD, coronary artery disease; CI, confidence interval; COPD, chronic obstructive pulmonary disease; TC, total cholesterol; HDL, high density lipoprotein; LDL, Low Density Lipoprotein; TG, triglyceride; IL-1β, Interleukin-1β; TNF-α, Tumor Necrosis Factor-α; IL-6, Interleukin-6; CRP, C-reactive protein.

### Examining the interactions between Metrnl with fatty acid levels and inflammatory cytokines

**Figure 1:**
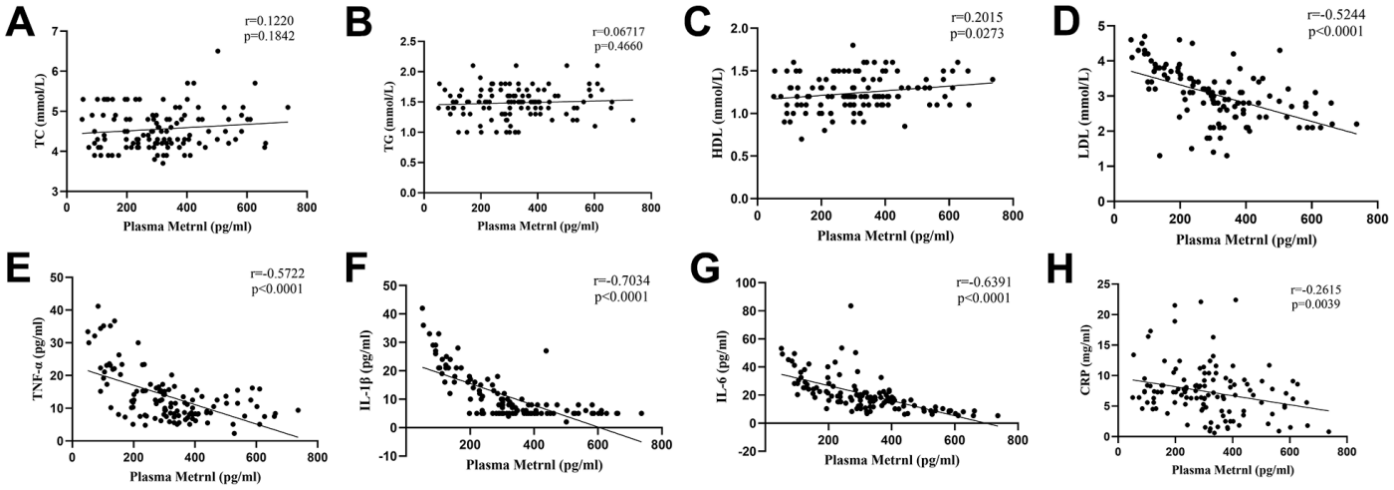
**Associations between plasma meteorin-like protein (Metrnl) levels, fatty acids, and inflammatory cytokines.** No significant correlation was present between Metrnl and (**A**) total cholesterol (TC), as well as (**B**) triglyceride (TG) levels. (**C**) Metrnl was positively correlated with high-density lipoprotein cholesterol (HDL). Metrnl was negatively correlated with (**D**) low-density lipoprotein cholesterol (LDL), (**E**) tumor necrosis factor (TNF)-α, (**F**) interleukin (IL)-1β, (**G**) IL-6, and (**H**) C-reactive protein (CRP). *P*<0.05 was statistically significant.

We then examined whether any relation between plasma Metrnl level and fatty acids, as well as inflammatory cytokines, were present. We found that no significant association was present between Metrnl and TC (Figure 1A), as well as TG levels (Figure 1B). However, Metrnl was positively associated with HDL (Figure 1C), and inversely associated with LDL levels (Figure 1D). As for inflammatory cytokines, inverse correlations were found between Metrnl and TNF-α (Figure 1E), IL-1β (Figure 1F), IL-6 (Figure 1G), and CRP (Figure 1H). These findings are consistent with the possibility that increased Metrnl levels may be linked to lowered inflammation and LDL levels.

### Lower Metrnl levels were associated with greater likelihood and severity of CAD

**Figure 2:**
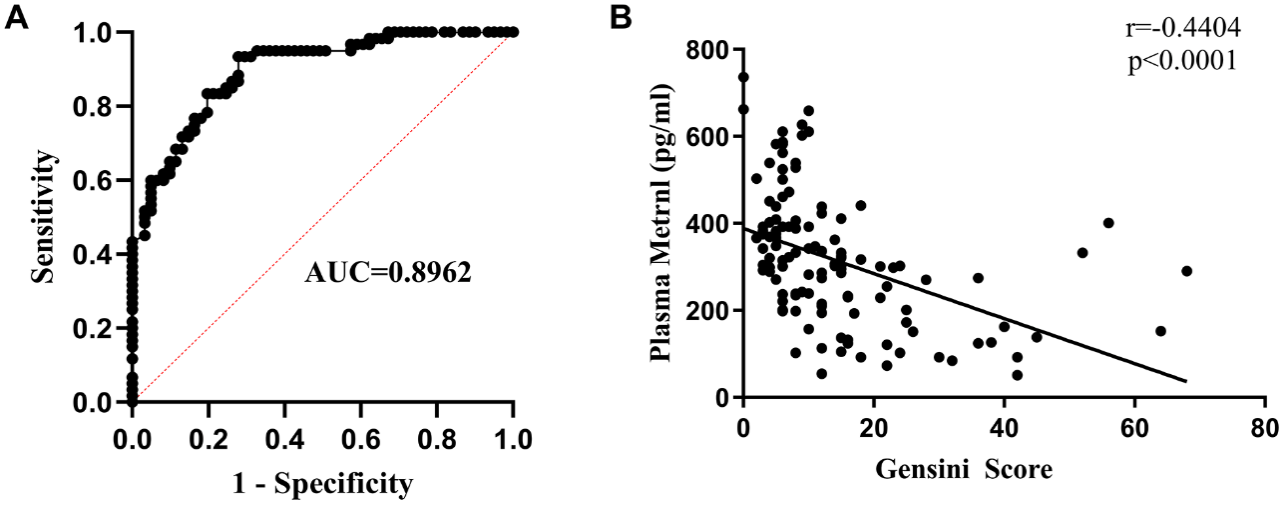
**Lower Metrnl levels were associated with greater likelihood and severity of coronary artery disease (CAD).** (**A**) Receiver operating characteristic (ROC) curve analysis demonstrating the accuracy of plasma Metrnl levels for diagnosing CAD. (**B**) Pearson correlation analysis demonstrating that higher Metrnl levels were inversely related to CAD severity, as defined by Gensini score.

To examine the relationship between Metrnl levels and CAD occurrence, multiple logistic regression analyses were carried out, where it was found that lowered Metrnl were significantly associated with increased likelihood for developing CAD. This was further supported by receiver operating characteristic (ROC) curve analysis, which demonstrated that Metrnl levels were highly accurate for diagnosing CAD presence (AUC=0.8962; Figure 2A). To determine the relationship between Metrnl and CAD severity, Pearson correlation analysis was used, and higher Metrnl levels were found to be inversely correlated with CAD severity, as defined by the Gensini score (Figure 2B). Therefore, lower Metrnl levels were found to correspond with increased likelihood and severity for CAD.

### Exercise-based rehabilitation corresponded with increased plasma Metrnl and reduced inflammation among CAD patients

**Figure 3:**
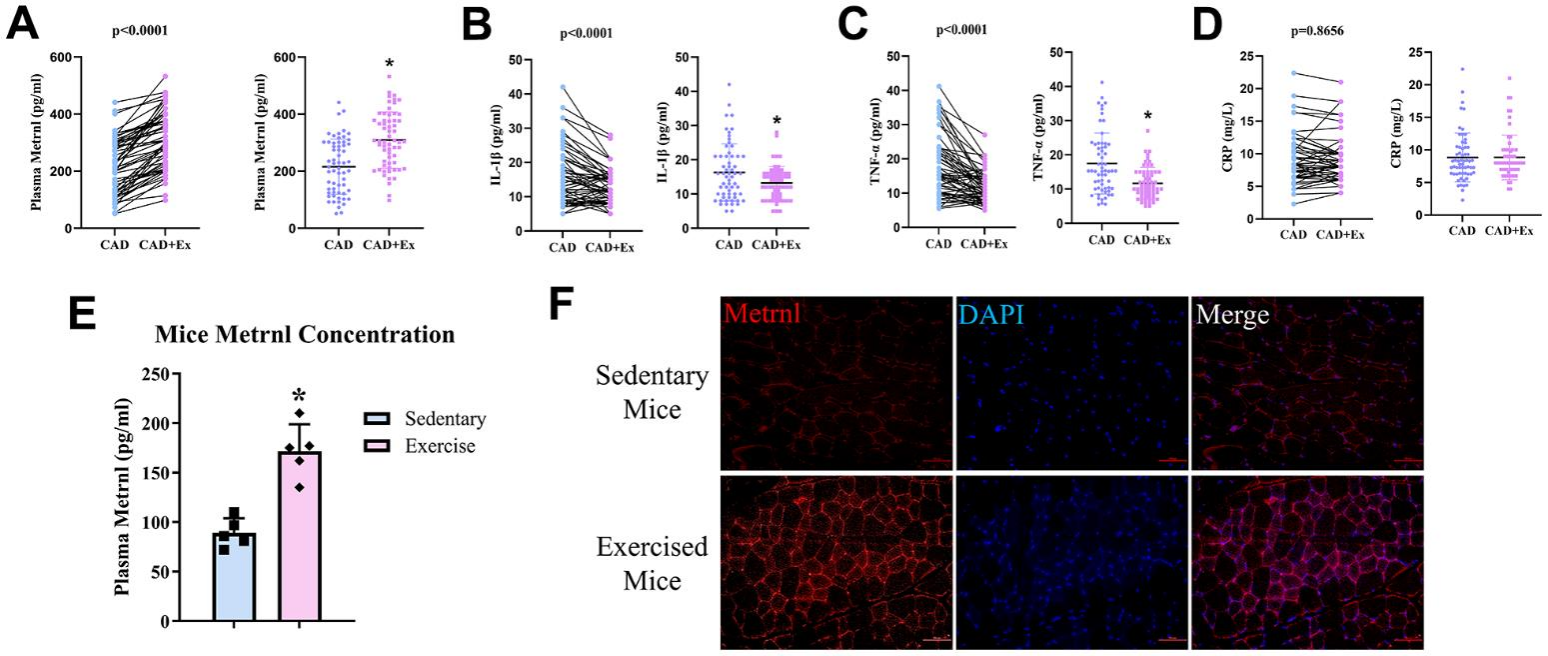
**Moderate-intensity continuous training (MICT) exercise was associated with increased plasma Metrnl and reduced inflammation.** (**A**) Plasma Metrnl levels increased among CAD patients who underwent the 12-week MICT exercise period (CAD+Ex), compared to those who did not (CAD group). Exercise was inversely associated with (**B**) IL-1β, (**C**) TNF-α, and (**D**) CRP levels. (**E**) Plasma Metrnl levels increased among mice who underwent the 12-week exercise period, compared to those who remained sedentary. (**F**) Immunofluorescence staining for Metrnl in skeletal muscle from sedentary and exercise mice groups. Results are shown as mean ± SD. N =5/group. * P<0.05 was statistically significant.

We then examined whether rehabilitative approaches, such as exercises, after CAD, could influence plasma Metrnl, as well as inflammatory cytokine levels. MICT was carried among CAD patients for 12 weeks, and Metrnl levels were measured before and after the 12-week exercise period. Indeed, Metrnl levels significantly increased after the exercise period (CAD+Ex), compared to pre-exercise (CAD; Figure 3A). On the other hand, IL-1β (Figure 3B), and TNF-α (Figure 3C) levels significantly decreased in CAD+Ex, compared to CAD, groups.

To further confirm the association between exercise and elevated Metrnl levels, we established a mouse exercise model, and compared plasma Metrnl levels between sedentary mice versus mice subject to exercises during a 12-week period. The results showed that mice subject to exercise had significantly higher Metrnl levels, compared to the sedentary group (Figure 3E). Immunofluorescence staining for Metrnl was then used to determine whether skeletal muscle contraction was the basis for increased Metrnl expression. Metrnl was observed to be localized within skeletal muscle fibers, and higher levels were present after exercise (Figure 3F). Thus, exercise was demonstrated to be an effective measure for increasing Metrnl, as well as decrease inflammation, in both CAD patients and mouse models.

### Metrnl alleviated palmitic acid-induced endothelial metabolic dysfunction by increasing aerobic respiration and reducing oxidative stress

**Figure 4:**
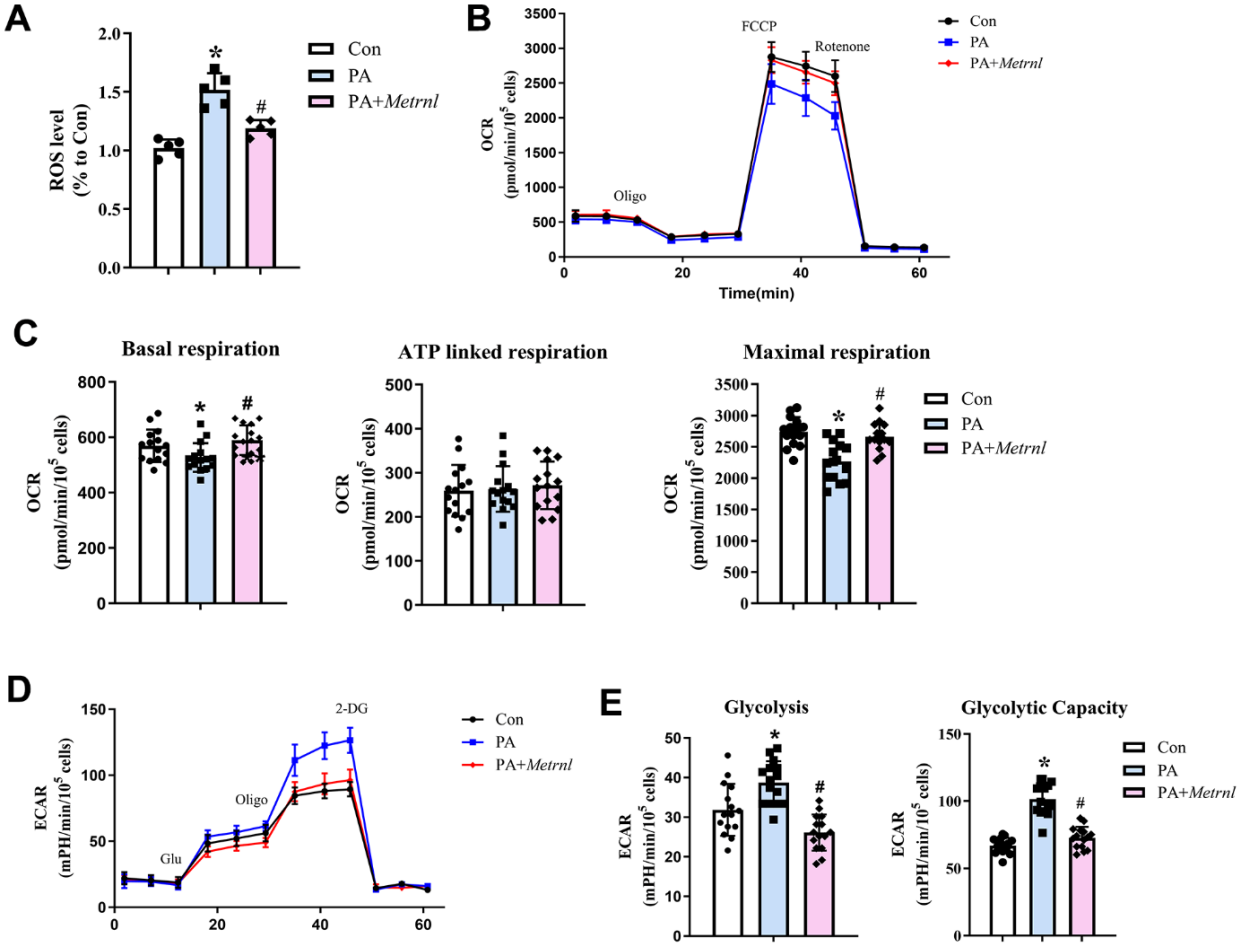
**Metrnl alleviated palmitic acid (PA)-induced metabolic dysfunction among human umbilical cord vein endothelial cells (HUVECs) by increasing aerobic respiration and reducing oxidative stress.** (**A**) Reactive oxygen species (ROS) levels among control (Con), PA-treated (PA), and co-treated PA+Metrnl (PA+Metrnl) HUVECs. (**B**) Representative traces of oxygen consumption rate (OCR) measurements for the 3 HUVEC groups through different ports in the flux analyzer: oligomycin, carbonyl cyanide 4-(trifluoromethoxy) phenylhydrazone (FCCP) and rotenone. (**C**) Quantification of basal, ATP-linked, and maximal respiration rates, derived from OCR measurements, for all 3 groups. (**D**) Representative traces of extracellular acidification rate (ECAR) measurements through different ports in the flux analyzer: glucose, oligomycin and 2-deoxy-glucose (2-DG). (**E**) Quantification analysis of glycolysis and glycolytic capacity, derived from ECAR measurements, for all 3 groups. Results are shown as mean ± SD. N =5/group. * P<0.05 was statistically significant.

The negative association found between plasma Metrnl and LDL among CAD patients suggested that Metrnl may also alleviate the negative impact of fatty acids on CAD. To explore the role of Metrnl on fatty acid-mediated effects in vascular tissue, palmitic acid was used to induce endothelial dysfunction among HUEVCs. The results showed that palmitic acid (PA)-treated HUVECs, compared to untreated controls (Con), had significantly higher ROS levels. However, these levels decreased towards that of the control group among PA HUVECs co-treated with Metrnl (PA+Metrnl; Figure 4A). To examine the impact of Metrnl application on aerobic oxidative metabolism, OCR was measured, using the XF24 extracellular flux analyzer. All 3 groups demonstrated similar OCR patterns upon oligomycin, FCCP, and rotenone/antimycin A administration, representing, respectively, glucose-linked (basal), ATP-linked, and maximal respiration measurements (Figure 4B). However, within these 3 groups, for basal and maximal respiration, PA had significantly lower OCRs, compared to Con and PA+Metrnl. OCR for ATP-linked respiration, by contrast, was the same among the 3 groups (Figure 4C). Therefore, Metrnl administration could reverse the PA-induced reduction in aerobic respiration. As for anaerobic respiration, or glycolysis, represented by ECAR, similar patterns were observed for all 3 groups upon glucose administration, representing glycolysis, as well as oligomycin and 2-deoxyglucose, representing glycolytic capacity (Figure 4D). However, unlike with OCR, ECAR was significantly higher for both glycolysis and glycolytic capacity in PA, compared to Con and PA+Metrnl, indicating that PA favors anaerobic respiration. This increase, however, is reversed back towards Con levels among PA+Metrnl (Figure 4E). This result thus demonstrated that increased Metrnl reduced anaerobic respiration by supporting more efficient pro-aerobic respiration among HUVECs.

### Metrnl alleviated palmitic acid-induced endothelial inflammatory responses via downregulating the ROS-NLRP3 signaling pathway

**Figure 5:**
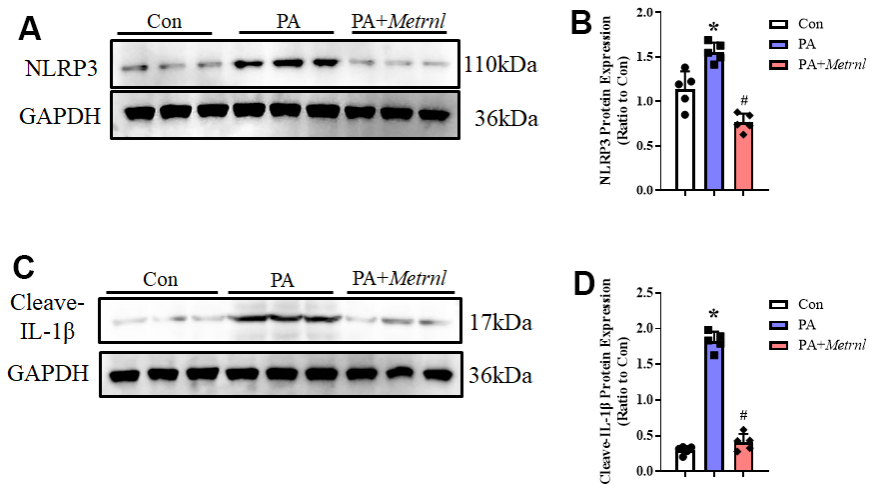
**Increased Metrnl alleviated PA-induced endothelial inflammatory responses via downregulating the ROS- NLR family pyrin domain containing 3 (NLRP3) signaling pathway.** (**A**, **B**) Representative Western blot images of NLRP3 levels and normalized to GAPDH among PA-treated HUVECs, with or without Metrnl co-treatment. (**C**, **D**) Representative Western blot images of IL-1β levels and normalized to GAPDH among PA-treated HUVECs, with or without Metrnl co-treatment. Results are shown as mean ± SD. N =5/group. *P < 0.05 vs Con, #P < 0.05 vs PA. P<0.05 was statistically significant.

Based on our findings that administering Metrnl to PA-treated HUVECs reduced ROS levels, we postulated that Metrnl may counteract against subsequent ROS effects, such as NLRP3 inflammasome activation. ROS has previously been demonstrated to be a significant trigger behind the activation of this inflammasome. Therefore, we investigated NLRP3 pathway activity in PA-treated HUVECs, with or without Metrnl treatment. Western blot analysis showed that NLRP3 and IL-1β were upregulated after PA, though the expression levels for both proteins decreased after Metrnl administration (Figure 5A, 5B). These observations thus suggested that Metrnl was able to reduce ROS levels, and subsequently downregulate NLRP3 activity, yielding reduced endothelial inflammatory cytokines.

## DISCUSSION

The association between exercise and quality of life improvements among CAD patients have long been observed, though the underlying basis behind these improvements have not been fully elaborated. One possible mechanism, though, may be increased Metrnl production from skeletal muscles, which have been observed post-exercise in previous studies [18]. This finding was supported by our observations in this study, in which plasma Metrnl levels were significantly higher among CAD patients who underwent a 12-week MICT exercise regime, compared to those who did not; the same findings were also found in a mouse model. These increased Metrnl levels, in turn, were associated with higher HDL and lower LDL, as well as decreased inflammatory cytokine expression, particularly TNF-α, IL-1β, and IL-6. These decreased cytokine levels were most likely due to NLRP3 inflammasome downregulation, as increased Metrnl was also associated with decreased ROS production and increased aerobic respiration among PA-treated HUVECs. Therefore, Metrnl is likely able to improve CAD patient functioning via increasing aerobic respiration, leading to downregulation of the ROS-NLRP3 signaling pathway, and subsequently lowered inflammatory cytokine production.

A connection between Metrnl and CAD have been recently documented in a previous study from Liu et al., where serum Metrnl levels was inversely correlated with CAD risk and severity, as determined by the Gensini score, in a study of Chinese patients [19]. Circulating Metrnl levels are associated with complications during the acute phase of STEMI and independently predict a worse outcome [22]. However, the exact underlying basis behind this negative correlation has not been fully defined, though knocking out Metrnl has been found to increase blood TG and lower HDL levels in a mouse model, suggesting that Metrnl may counteract against CAD occurrence via influencing lipid metabolism [23]. The findings from the mouse model were also supported by the findings in our study, in which increased Metrnl was positively associated with HDL, but negatively associated with LDL levels among CAD patients. In fact, Metrnl was first identified as an adipokine produced by white adipose tissue [24], where it was involved in the “browning” of white adipose to brown adipose [15]. With respect to lipid metabolism, Metrnl was also found to upregulate genes involved in lipid transport, lipogenesis, lipolysis, and storage [15], along with being able to enhance serum TG clearance during an acute lipid overload test [15]. It is also possible that Metrnl could be involved in reducing lipoprotein cholesterol levels directly [25]. All of these factors could thus serve as a possible basis behind exercise being associated with improved quality of life among CAD patients, as exercise has been documented to be associated with increased Metrnl, and subsequently increased fatty acid uptake and oxidation in skeletal muscle [26], particularly via AMP-activated protein kinase (AMPK) or peroxisome proliferator-activated receptor (PPAR)δ-dependent signaling pathways. These pathways, in turn, increased the expression of fatty acid oxidation-linked genes, such as fatty acid binding protein (FABP)3, which also falls in the same fatty acid binding protein family as the FABP4 documented in adipose tissue, further reinforcing the connection between Metrnl and fatty acid metabolism [15, 26]. Metrnl production during exercise has been documented to stem from skeletal muscles in numerous studies [15, 26–28], possibly via the activation of the PGC-1α-4 isoform. More specifically, exercise increases the activity of signaling pathways, such as AMPK, which then activate PGC-1α-4, in turn increasing Metrnl production [26]. In addition, some studies found that resistance exercise, which may be related to skeletal muscle loss in diabetic patients [29]. Metrnl may be a future effective treatment for improving exercise resistance in diabetic patients. In line with this proposed physiological process, we found, under immunofluorescence staining, increased Metrnl expression among skeletal muscles from mice who were subjected to a 12-week exercise period, compared to those who remained sedentary. This increased Metrnl production, in turn, not only increases fatty acid metabolism, but also glucose metabolism among skeletal muscles, via improving glucose uptake. More specifically, Metrnl is involved in activating multiple signaling pathways to increase glucose transporter type 4 (GLUT4) expression, such as AMPK/mitogen-activated protein kinase (MAPK) and histone deacetylase 5 (HDAC5) [26].

The association between increased Metrnl and greater aerobic respiration was also in accordance with our findings from PA-exposed HUVECs, in which HUVECs co-treated with Metrnl had OCR levels comparable to that of untreated HUVECs, and higher than that of PA-treated ones, as well as ECAR levels comparable to untreated HUVECs, and lower than that of PA-treated cells, indicating improved aerobic respiration compared to the latter HUVEC group. ECAR is a measure of lactic acid levels, generated by anaerobic glycolysis. In our study, we found that OCR enhanced and ECAR decreased under Metrnl treatment, demonstrating that aerobic metabolism was more active than before and consumed more pyruvate. This association between PA and reduced aerobic respiration, and Metrnl being able to counteract against these negative impacts, could serve as a basis behind the association of obesity with decreased fatty acid and glucose metabolism, and subsequently higher CAD likelihood [26].

Endothelial dysfunction is one of the first recognizable signs of atherosclerosis, and also contributes to the progression of atherosclerosis by various mechanism [30]. Regular physical exercise improves both basal endothelial nitric oxide formation and endothelium-dependent vasodilation of the skeletal muscle vasculature in patients [31]. In our research, aerobic exercise reduced oxidative stress and improved endothelial energy metabolism and endothelial dysfunction by increasing serum Metrnl level. Indeed, obese individuals have been observed to have lower serum Metrnl levels [28], further reinforcing the underlying mechanistic basis of exercise being able to improve quality of life among CAD patients via increasing Metrnl production, leading to increased fatty acid and glucose metabolism, via activating pro-metabolic signaling pathways mediated by AMPK and PPARδ [18]. These signaling pathways also served as a basis behind the link between Metrnl and the production of pro-inflammatory cytokines. More specifically, Metrnl has been documented to be associated with lowered pro-inflammatory cytokine production, such as TNF-α and IL-6 [18, 32], which has also been observed in our study among CAD patients who underwent exercises during a 12-week period, compared to pre-exercise levels. This could be the basis behind the finding that decreased Metrnl levels were present among individuals with type 2 diabetes and obesity, both of which have been considered as “low-level inflammatory conditions” [23, 28]. Inflammation has also been linked with worsening CAD outcomes [33], no matter the cholesterol levels present [34]. This is further reinforced with the finding from a study of 70 CAD patients subjected to exercise, in which exercise was found to reduce inflammation, compared to those who did not undergo such regimens [3]. Furthermore, numerous genes have been identified from genome-wide association studies to link inflammation with CAD occurrence. In fact, one such gene identified was IL6-R, the receptor for IL-6, which is in line with our findings that decreased Metrnl was linked with increased IL-6 production, as well as being associated with the NLRP3 inflammasome [35].

As for NLRP3, it has previously been linked to cardiovascular diseases, particularly atherosclerosis, which is a significant causative mechanism behind CAD. For instance, cholesterol crystals and oxidized LDL have been found to activate the inflammasome among mouse and human macrophages, leading to the production of inflammatory cytokines, such as IL-1β [36]. These cytokines, in turn, contribute to the formation of the unstable atherosclerotic plaque via recruiting monocytes, increasing the permeability of the endothelial cell layer, promoting differentiation of arterial smooth muscle cells, facilitating apoptosis, and triggering intra-plaque hemorrhage via activating vascular endothelial growth factor [37]. Rupturing of the plaque, in turn, results in coronary vessel-occluding thrombi being formed, leading to CAD [38]. Therefore, this mechanism where NLRP3 inflammasome activation serves as the underlying basis between increased fatty acid levels, particularly LDL, being associated with higher CAD risk, is supported by our findings that CAD patients had increased LDL and inflammatory cytokines IL-1β, IL-6, TNF-α, and CRP, compared to non-CAD ones. Another factor that could increase NLRP3 activity is ROS, which has been found to be linked to the final activation of the inflammasome [36]. This observation is also in line with our findings that lowered ROS production was found among PA-exposed HUVECs after co-treatment with Metrnl, compared to those who did not receive Metrnl. Furthermore, both NLRP3 inflammasome and IL-1β expression levels were upregulated among PA-exposed HUVECs, and these levels decreased after Metrnl co-treatment, further validating the link between increased ROS, NLRP3 activity, and inflammatory cytokine production, all of which have been associated with lowered functional capabilities in CAD. As a result, Metrnl is able to decrease ROS levels via promoting aerobic respiration, leading to reduced NLRP3 activation and inflammatory cytokine production, and thus subsequently alleviating the negative impacts of CAD.

## CONCLUSIONS

We found that CAD patients who underwent MICT exercises had increased skeletal muscle Metrnl production, compared to those who did not. Furthermore, higher Metrnl levels were associated with decreased levels of LDL, as well as inflammatory cytokines IL-1β, IL-6, TNF-α, and CRP, all of which were linked to CAD onset. These correlations were reinforced by higher Metrnl levels also corresponding to lessened CAD risk and severity, which may be based on Metrnl being able to counteract against ROS production. Lowered ROS, in turn, downregulated NLRP3 inflammasome activity and subsequent inflammatory cytokine production, as well as improving endothelial aerobic glucose metabolism. Therefore, Metrnl may serve as a potential therapeutic agent for improving CAD patient functioning and quality of life.

## MATERIALS AND METHODS

### Study population and exercises

One hundred twenty individuals who passed through the Department of Cardiology at Shenzhen People’s Hospital, between March-October 2021, were randomly recruited. These individuals were divided into 2 groups of 60 patients each: control and CAD. Control patients were defined as atherosclerotic patients with <50% coronary stenosis, as observed under coronary angiographs, while CAD patients had >50% coronary stenosis. Patients from both groups were age- and gender-matched. CAD patients were then subjected to MICT exercises, comprising continuous cycling at 45–60% of heart rate reserve, for 30–45 min, using a stationary bicycle, with ergometer SCHILLER CS-200 attached, for 12 weeks; each week comprised of 3 exercise sessions. Additionally, from all patients, 10 mL of peripheral blood were collected, and all subsequent analyses were normalized to hematocrit to account for dilutions. The study was approved by the Committee for Medical and Health Ethics of Shenzhen People’s Hospital, Jinan University, in accordance with the Declaration of Helsinki. All patients provided written informed consent.

### Cell culture

Human umbilical vein endothelial cells (HUVECs) were purchased from the American Type Culture Collection (Manassas, VA, USA), and cultured using Dulbecco’s modified Eagle’s medium containing 10% fetal bovine serum, 50 U/mL penicillin, and 50 mg/mL streptomycin (Invitrogen, Carlsbad, CA, USA).

### Plasma Metrnl and inflammation cytokine measurements using ELISA

Human patient and mouse blood samples by tail incision were collected immediately after exercise and centrifuged at 3000 rpm for 10 min at 4° C. Either a human or mouse Metrnl ELISA kit was used to measure plasma Metrnl levels, according to the manufacturer’s instructions (both R&D systems), while human ELISA kits were used to measure inflammatory cytokines (Cat #: ab181421, ab46052, ab178013, Abcam, USA).

### Exercise training for mice

To adapt the mice for exercise training, mice were trained at 1 week prior to the 12-week exercise period on a specially-designed mouse treadmill. The treadmill speed was initially set at 0.5 km/h. After adaption, the speed gradually increased to 0.6, 0.7, 0.8, and 1.0 km/h(maximum), for 1 h, on subsequent consecutive days.

### Immunofluorescent staining of mouse skeletal muscle tissue sections

To carry out immunofluorescence staining, mouse skeletal muscle tissue sections were fixed in 4% paraformaldehyde, blocked with 10% goat serum, and incubated with anti-mouse Metrnl primary antibody overnight at 4° C (1:100; Cat #: bs-18810R, Bioss, USA). The sections were then incubated with Alexa Fluor 546-conjugated secondary antibodies (1:400; Invitrogen, USA), nuclei stained with 4′,6-diamidino-2-phenylindole (DAPI) for 5 min, and images taken using a confocal microscope (LSM 780, Zeiss, Germany).

### Measuring mitochondrial ROS levels

To measure mitochondrial ROS levels, HUVECs were stained with MitoTracker Red (0.5 mM; excitation/emission 550/590 nm, Invitrogen), and superoxide levels were examined according to changes in MitoSOX Red fluorescence using a Zeiss confocal microscope (LSM 780). Mean fluorescence values were analyzed by ImageJ, and compared to untreated control HUVECs.

### Analysis of oxidative and glycolytic metabolic rates

Oxygen consumption (OCR) and extracellular acidification (ECAR) rates for HUVECs were measured using, respectively, the Seahorse XF Mito and Glycolysis Stress Test Kits on an XF24 Extracellular Flux Analyzer (Agilent Technologies, USA). HUVECs were plated 1 day prior to metabolic analysis, and metabolic rates were measured in the absence (basal conditions) or presence of inhibitors. For OCR, the following compounds were sequentially added into the cells through different ports in the flux analyzer: oligomycin (ATP synthase inhibitor; 1 μM), carbonyl cyanide 4-(trifluoromethoxy) phenylhydrazone (mitochondrial uncoupling agent FCCP; 1 μM) and rotenone (mitochondrial complex inhibitor; 1 μM), while for ECAR, instead of FCCP and rotenone, 2-deoxy-glucose (2-DG, glucose analog serving as a hexokinase inhibitor; 5 mM) was added after glucose and oligomycin. The resulting OCR and ECAR were normalized to basal respiration rates.

### Western blotting

Total protein samples (80 μg) were subjected to electrophoresis in 4%-15% SDS-PAGE gel, followed by transfer into polyvinylidene difluoride membranes, which were then blocked with 5% casein at room temperature for 1 h. Primary antibody incubation for NLRP3 (Cat #15101S, Cell Signaling Technology), IL-1β (Cat #2022, Cell Signaling Technology, USA), and GAPDH (Cat #2118, Cell Signaling Technology, USA) were carried out (all 1:1000) overnight at 4° C. Afterwards, membranes were incubated with horseradish peroxidase-conjugated secondary antibody (1:2000) for 90 min at room temperature. After being washed 3 times, membranes were then subjected to enhanced chemiluminescence detection (WesternBright). Images were captured using ChemiDoc XRS system (Bio-Rad, USA) and analyzed by ImageJ.

### Statistical analysis

Statistical analyses were performed by SPSS ver. 20.0. Data are expressed as mean ± standard deviation (SD), except for patient characteristics between control and CAD groups, which were expressed either as mean values with 95% confidence intervals (CI), or as percentages. To evaluate the difference between two groups, we used the χ2 analysis for categorical variables, the Student’s *t*-test for continuous variables and multiple logistic regression test for nominal variables. Receiver operating characteristic (ROC) curve analysis was used to determine the optimum cut-off level for Metrnl in predicting CAD occurrence, while Pearson correlation was used to examine the presence of associations between Metrnl with inflammatory cytokines, Total Cholesterol (TC), Triglycerides (TG), HDL, LDL, and Gensini scores. Student’s *t*-test was used for comparisons between 2 groups, while one-way analysis of variance (ANOVA), followed by Bonferroni *post hoc* tests. *P*<0.05 was considered statistically significant.

### Data availability statement

Raw data supporting the conclusions of this article will be made available by the authors, without undue reservation.
